# Partial differential equations in data science

**DOI:** 10.1098/rsta.2024.0249

**Published:** 2025-06-05

**Authors:** Andrea L. Bertozzi, Nadejda Drenska, Jonas Latz, Matthew Thorpe

**Affiliations:** ^1^ Department of Mathematics, UCLA, Los Angeles, CA, USA; ^2^ Department of Mathematics, Louisiana State University, Baton Rouge, LA, USA; ^3^ Department of Mathematics, The University of Manchester, Manchester, UK; ^4^ Department of Statistics, University of Warwick, Coventry, UK

**Keywords:** machine learning, partial differential equations, deep neural networks

## Abstract

The advent of artificial intelligence and machine learning has led to significant technological and scientific progress, but also to new challenges. Partial differential equations, usually used to model systems in the sciences, have shown to be useful tools in a variety of tasks in the data sciences, be it just as physical models to describe physical data, as more general models to replace or construct artificial neural networks, or as analytical tools to analyse stochastic processes appearing in the training of machine-learning models. This article acts as an introduction of a theme issue covering synergies and intersections of partial differential equations and data science. We briefly review some aspects of these synergies and intersections in this article and end with an editorial foreword to the issue.

This article is part of the theme issue ‘Partial differential equations in data science’.

## 1. Introduction

The analysis of data is a major scientific, industrial and societal challenge. Modern data analysis is highly diverse: evaluation of survey data that influences political decisions, the segmentation of medical images used for diagnoses, the assimilation of data into a weather model for accurate predictions or the recognition of the contour of a face to unlock someone’s smartphone. Some of these challenging problems have profited from the recent advent of machine-learning methods and artificial intelligence (AI).

Machine learning and AI come with many advantages, but also challenges: be it mathematical, computational, environmental or from a safety perspective.

Partial differential equations (PDEs) have recently gained popularity in the realm of data science. PDEs are usually used in the physical, biological or social sciences to model the behaviour of systems or processes of interest. To name a few: *heat equations* describe the temperature distribution in a solid object, *Navier–Stokes equations* describe fluid flow or *Schrödinger equations* are fundamental in quantum mechanics. Traditionally, PDEs and data science meet when data shall be used to calibrate parameters in the PDEs.

Today, PDEs play a much broader role within data science: PDEs are used as machine-learning models, they are used to construct architectures of artificial neural networks, they are used to analyse stochastic processes that appear within machine learning and so on. This article forms the introduction of a theme issue in the *Philosophical Transactions of the Royal Society A* titled ‘Partial differential equations in data science’. The theme issue collects current research on intersections and synergies between data science and PDEs. This article acts as a brief review of some of these intersections and synergies. We give a detailed outline next.

### (a) Outline, limitations and topics not included

In this article, we will give a brief introduction to three distinct areas in which PDEs interact with data science.

Section 2 discusses Bayesian and variational methodology used to blend PDEs and data.

Section 3 introduces PDEs that are used as machine-learning models.

Section 4 handles topics at the intersection of PDEs and artificial neural networks.

This introductory article ends with a foreword to the theme issue in Section 5.

Data science is a very fast-moving field, and this article clearly cannot cover all aspects of PDEs in data science. Sections 2–4 act as an introductory overview into ways PDEs and data science interact, but do not intend to be exhaustive presentations of their subjects. We now list a few intersections of PDEs and data science that are not covered elsewhere in this article.

*Optimal transport*: Through gradient flows optimal transport connects with PDEs [1]. Within data science, optimal transport plays a role as an interpretable metric that often captures the underlying geometry better by modelling translations [2]. Optimal transport has been particularly useful for distribution matching: a problem that arises naturally in generative adversarial networks (GANs); see in particular the Wasserstein GAN [3].

*Generators of stochastic processes*: Stochastic processes appear frequently in data science, be it as optimization [4] or sampling methods in training procedures or as generative models [5]. Processes that are defined in continuous time can often be analysed through their infinitesimal generators, which are often given as differential operators.

*Physics-informed machine learning*: When neural networks are supposed to represent the behaviour of physical systems, they are sometimes trained to satisfy certain physical laws. This topic was covered in Volume 381, Issue 2260 and Volume 382, Issue 2264 of the *Philosophical Transactions of the Royal Society A*.

*Operator learning*: In operator learning, the goal is to identify an operator from input–output pairs. Often these operators are solution operators of parametric PDEs (e.g. [6,7]) or transfer operators of dynamical systems (e.g. [8,9]).

## 2. Blending partial differential equations and data

When a mathematical model is used to describe a particular system or process in, e.g. the physical or social sciences, it is often necessary to first calibrate the model with respect to observational data. Mathematical models are usually given in fairly general settings and come with a set of parameters that allow the applicant to adapt the model to a variety of real-world scenarios. Given observational data, the problem is then to find a combination of parameters with which the model describes real-world scenarios in which the data have been collected. Other than *calibration*, this process is often referred to as an *inverse problem*. A related problem when considering dynamical systems is that the parameters of the system may be known, but its current state is not known and needs to be estimated. This is especially critical if the problem is stochastic or chaotic. The process of calibrating a model by continuously estimating and forecasting its state is called *data assimilation*. In the following, we focus on the inverse problem, and we refer to [10,11] for details on data assimilation.

In the context of mathematical models given through solutions of PDEs, *parameters* can be scalars, vectors, functions, sets or else. States are usually vectors, functions or distributions. Their identification from observational data leads to a blending of PDE and data and is a fundamental task in data science. The task comes with mathematical, statistical and computational challenges. We describe some of these challenges and their solution below; we start with a general framework and an example.

### (a) Inverse problems

We first present a usual set-up for inverse problems in PDEs. We often assume to have


$$G(\theta^{†})+\eta^{†}=y^{†},$$

where $\theta^{†}$ is the unknown parameter, $y^{†}$ is observational data given through a parameter-to-data map G and perturbed by observational noise $\eta^{†}$ . The data live in a data space Y , which is usually finite-dimensional; the parameter lives in a parameter space X . We usually think of the operator G:X→Y as given through a PDE. The inverse problem now consists in identifying the parameter from the observational data. Doing so is challenging: the operator G is usually nonlinear, it often needs to be approximated by a numerical method and it usually is not invertible or not boundedly invertible. The latter two points lead to *ill-posedness*: the problem has no solution, no unique solution or the solution does not depend continuously on the data [12].

Bayesian and variational strategies have been employed in the past to counteract this ill-posedness. In the following sections, we will discuss these approaches after giving a motivational example.

***Example. (Darcy flow).*** Our goal is to represent the pressure head in a groundwater reservoir $D\subset {\mathbb{R}}^{3}$ —open, bounded and connected. At stationarity, this pressure head is a function p:D→ℝ . Following Darcy’s Law, p can be described by an elliptic PDE


∇⋅(θ(x)∇p(x))=f(x)(x∈D),

where θ:D→(0,∞) describes the permeability of the rock in the reservoir, f:D→ℝ is a source term and p is subject to appropriate boundary conditions on ∂D . While we usually assume the source term f and boundary conditions are known, the permeability θ is hidden underground and needs to be estimated. In hydraulic tomography [13,14], this problem usually consists of identifying θ from noisy pressure measurements at certain points in the reservoir. Hence, we have


$$G(\theta )=(p(x_{1}),\ldots ,p(x_{n})),$$

where $x_{1},\ldots ,x_{n}\in D$ are the distinct spatial measurement locations. This inverse problem has been studied extensively from a mathematical, statistical and computational perspective (e.g. [15–17]).

### (b) Bayesian inverse problems

The Bayesian approach to inverse problems is based on the Bayesian statistical paradigm. The paradigm goes back to Bayes [18] and Laplace [19]. Early works on Bayesian methodology in the context of PDEs and inverse problems are [20–22].

The idea of the Bayesian paradigm is that the unknown parameter $\theta^{†}$ is represented by a random variable θ . This random variable is distributed according to a so-called *prior distribution* $\mu_{\text{prior}}$ , which encodes uncertainty present in the parameter before the data are observed. The goal is now to condition this random variable on the observed data, that is, to find the distribution of θ given that $y^{†}$ has been observed. In particular, we assume that the noise is given by a random variable $\eta ∼\mu_{\text{noise}}$ , and then we aim to find the *posterior distribution*


$$\mu_{\text{post}}^{†}=P(\theta \in \cdot |G(\theta )+\eta =y^{†}).$$

This posterior distribution can often be found by Bayes’ theorem. Assume, for instance, that $\mu_{\text{prior}}$ and $\mu_{\text{noise}}$ have associated probability density functions $\rho_{\text{prior}}$ and $\rho_{\text{noise}}$ . Then, Bayes’ theorem states that $\mu_{\text{post}}^{†}$ has a density $\rho_{\text{post}}^{†}$ , which is given by


$$\rho_{\text{post}}^{†}(\theta )=\frac{\rho_{\text{noise}}(y^{†}-G(\theta ))\rho_{\text{prior}}(\theta )}{\int_{X}\rho_{\text{noise}}(y^{†}-G(\theta^{\prime }))\rho_{\text{prior}}(\theta^{\prime })d\theta^{\prime }}\;(\theta \in X).$$

In practice, the posterior is usually approximated using Markov chain Monte Carlo methods. Several strategies have been proposed to cope with high- and infinite-dimensional parameter spaces in Bayesian inverse problems (e.g. [23–25]), as well as with the computational burden from the repeated PDE discretizations (e.g. [26–29]).

Bayesian inverse problems are well-posed under very weak assumptions (e.g. [21,30–32]). Another advantage of the Bayesian approach to inverse problems is that it allows the applicant to quantify uncertainties that remain in the PDE-based model after seeing the data. Indeed, when employing the Bayesian approach to inverse problems to the Darcy flow problem, the elliptic PDE turns into a stochastic PDE with random diffusion coefficient. The latter is not true in the variational approach to inverse problems, which we discuss next.

### (c) Variational inverse problems

Starting from the Bayesian approach to inverse problems induced by the Bayesian paradigm, variational inverse problems have a stronger connection to classical statistics and point estimation. The fundamental idea is that we aim to find the parameter by maximizing the likelihood, i.e. we compute


$$\min_{\theta \in X}-\log\rho_{\text{noise}}(y^{†}-G(\theta )),$$

as usual in parametric statistics. If $\rho_{\text{noise}}$ is non-degenerate Gaussian, this turns into a nonlinear least squares problem, i.e.


$$-\log\rho_{\text{noise}}(y^{†}-G(\theta ))=\frac{1}{2}\|y^{†}-G(\theta ){\|}_{*}^{2},$$

for an appropriately weighted 2-norm $\|\cdot {\|}_{*}$ . Least squares problems of this form go back to Gauss and Legendre (e.g. [33]). Now, minimization problems of this type often have a solution, but they are likely still ill-posed. In variational inversion, ill-posedness is resolved by regularization (see [34,35] for an overview). A regularization method often employs a *regularizer* R:X→ℝ that is added to the target function mentioned above, i.e. one minimizes


$$\min_{\theta \in X}-\log\rho_{\text{noise}}(y^{†}-G(\theta ))+R(\theta ).$$

We have broadly motivated the first optimization problem as a maximum likelihood estimation idea. The variational problem here could then be seen as maximum *a posteriori* estimation, i.e. we try to find the maximum of a posterior density. The regularizer R then plays the role of the prior, although not every regularizer defines a prior distribution [36]. There are also other forms of regularization, e.g. early stopping of iterative optimization methods can be shown to have a regularizing effect [34].

A variety of regularizers have been proposed for variational inverse problems (e.g. [37–39]), and the well-posedness of the associated inverse problems has been studied. The regularized optimization problems tend to be difficult to solve: a nonlinear G may lead to non-convexity, regularizers are sometimes non-smooth and a large amount of data may be difficult to store in memory throughout the algorithm.

### (d) Priors and regularizers given by differential operators

The operator G in an inverse problem is not always given by a differential equation. In image reconstruction, for instance, integral operators are very common, e.g. in deconvolution problems or in X-ray tomography. In other situations, we may make noisy observations of an unknown function at certain spatial locations and try to recover the function in a non-parametric regression problem. Here θ is just the unknown function. In such situations, (partial) differential operators are often used to define prior distributions and regularizers. We now give a few examples for such priors and regularizers.

In the Bayesian setting, the prior measure of function-valued parameters can sometimes be given by Gaussian measures on function spaces. Gaussian measures with *Whittle–Matérn covariance operators* are particularly popular [40]. A sample θ from such a prior can be written as the solution to a certain stochastic PDE


$$(\kappa^{2}I-\Delta {)}^{\alpha /2}\theta (x)=B(x)\;(x\in R^{d}),$$

where α>d/2,κ>0 are parameters and B is spatial Gaussian white noise. This model is able to represent a large range of random functions: α controls the smoothness of the function, and $\kappa^{2}$ controls the correlation length. We refer to [41,42] for fundamental works regarding such Stochastic Partial Differential Equation models and to, e.g. [43] for an application.

In the variational setting, regularizers also contain differential operators to control the regularity of an unknown function-valued parameter $\theta :[0,1{]}^{d}\to \mathbb{R}$ . The *total variation functional*


$$\|\theta {\|}_{\text{TV}}:=\int_{[0,1{]}^{d}}\|\nabla \theta (x){\|}_{2}dx,$$

for instance, favours piecewise constant functions [44] and is a popular regularization technique. Similarly, total generalized variation produces piecewise smooth functions [45]. More regularity can be obtained using (fractional) Laplace operators [46,47], while binary functions are sometimes represented using diffusive interface models [48].

## 3. Partial differential equations as machine learning models

Machine learning can be broadly classified into *Supervised Learning*, *Semi-Supervised Learning, Unsupervised Learning* and *Reinforcement Learning* [49]. In this paper, we present examples related to PDEs of the former three. We start by introducing supervised, unsupervised and semi-supervised learning, before then moving on to related PDE models.

*Supervised Learning* [50] uses fully labelled datasets and trains models to be able to predict labels for unseen data. In particular, suppose there are n labelled points, $x_{1},\ldots ,x_{n}\in \Omega$ , whose labels $\ell_{1},\ldots ,\ell_{n}\in L$ are known (with some appropriate label space L ). A learning algorithm uses the labelled data $(x_{1},\ell_{1}),\ldots ,\left(x_{n},\ell_{n}\right)$ to find an underlying function $u^{†}:\Omega \to L$ , which associates any x∈Ω with an appropriate label ℓ . We construct a model or hypothesis u:Ω→R for $u^{†}$ . The model u should be aligned with the learning data $\{x_{i},\ell_{i}{\}}_{i=1}^{n}$ , but we can also ask for it to have other properties, such as smoothness. Usual models are polynomials, neural networks or Gaussian processes. The approach we discuss in the following is to find such a model u as a solution of a PDE, or as a solution to a model coming from a discrete version of a PDE. Some examples include ‘graph Laplacian’ techniques, described below.

*Unsupervised Learning* [50] finds patterns in unlabelled data. One example of unsupervised learning is clustering, which is used in various data-driven applications. Given n unlabelled points, $x_{1},\ldots ,x_{n}$ , the objective is to split them into groups, where points in each group are similar to one another. PDE-based methods can be used here if clusters are difficult to separate with basic algorithms, such as k-means.

*Semi-Supervised Learning* combines aspects of both unsupervised and supervised learning [51]. There are m<n labelled points, $x_{1},\ldots ,x_{m}$ , whose labels $\ell_{1},\ldots ,\ell_{m}$ are known, and n−m unlabelled points $x_{m+1},\ldots ,x_{n}$ . We let $\Omega_{n}=\{x_{i}{\}}_{i=1}^{n}\subset \Omega$ where typically $x_{i}\in {\mathbb{R}}^{d}$ for some d∈ℕ . The goal is to ‘learn’, or infer, a function u , with $u:\Omega_{n}\to L$ using information from both the labelled data $(x_{1},\ell_{1}),\ldots ,\left(x_{m},\ell_{m}\right)$ and the unlabelled data $x_{m+1},\ldots ,x_{n}$ . The unlabelled data help to better explore the geometry of the space Ω and to find a better model, especially in situations where only a small amount of labelled data are available.

### (a) The graph Laplacian

Many data-driven tasks in the areas of machine learning and data science use a notion of similarity to define a geometry via a graph structure. The vertices of the graph are the feature vectors $x_{1},\ldots ,x_{n}\in \Omega$ represented, for example, as points in ${\mathbb{R}}^{d}$ , and edges between nodes are weighted using some given similarity (which could be computed based on a distance between the vertices). In particular, we define an undirected graph G=(V,E) with vertices $V=\Omega_{n}$ and edges E⊆{{i,j}:i,j=1,…,n,i≠j} . In addition, E is also equipped with a symmetric weight matrix $W_{n}\in {\mathbb{R}}_{+}^{n\times n}$ with entries $w_{ij}$ . Intuitively, $w_{ij}\gg 0$ if $x_{i},x_{j}$ are similar and $w_{ij}\approx 0$ otherwise. Also, $w_{ij}=0$ if i=j or {i,j}∉E . We refer to [52] for different ways to construct such graphs and weight matrices.

Data occurring in natural settings often lie on a low-dimensional manifold that is approximated by this graph. Finding this manifold is of considerable interest, and learning methods related to this are called *manifold learning* [53] or *nonlinear dimensionality reduction*. In order to use this phenomenon, researchers have introduced *graph Laplacians* and investigated their properties; see, for example, [53–57] among others. Intuitively, these methods aim to identify the geometry of a space by characterizing diffusion on that space.

The (*unnormalized) graph Laplacian* is


$$L_{n}u(x_{i})=\sum_{j\in N_{i}}w_{ij}(u(x_{i})-u(x_{j})),$$

where $N_{i}$ denotes the neighbourhood of $x_{i}$ (that is, the set of indices ⊆{1,…,n} vertices $x_{j}$ that have positive weights $w_{ij}>0$ between themselves and $x_{i}$ ). When identifying $u:\Omega_{n}=\{x_{i}{\}}_{i=1}^{n}\to R$ with a vector $u\in R^{n}$ via $u(x_{i})=u_{i}$ , we can also write $L_{n}=D_{n}-W_{n}\in R^{n\times n}$ where $D_{n}\in R^{n\times n}$ is the diagonal matrix of degrees, i.e. $D_{n}=(d_{ij}{)}_{i,j=1}^{n}$ where $d_{ii}=\sum_{j=1}^{n}w_{ij},$ and $d_{ij}=0$ whenever i≠j , and $W_{n}$ is the weight matrix as given above. We sometimes consider normalized versions of the graph Laplacian, such as the random walk Laplacian or the symmetrically normalized Laplacian. The *Random Walk Laplacian*


$$L_{n}^{\left(r\right)}u(x_{i})=\frac{1}{\sum_{j\in N_{i}}w_{ij}}\sum_{j\in N_{i}}w_{ij}(u(x_{i})-u(x_{j}))$$

is the graph Laplacian, reweighted, so that the coefficient in front of $u(x_{i})$ is 1 . It is used in the analysis of random walks on graphs, as the coefficients


$$\frac{w_{ij}}{\sum_{j\in N_{i}}w_{ij}},$$

are the transition probabilities of a random walk on the weighted graph.

There is a whole class of Laplacians of the form ${\tilde{L}}_{n}=D_{n}^{1-p/q-1}(D_{n}-W_{n})D_{n}^{-r/q-1}$ that have been considered (see [58]). Of note is the *symmetrically normalized Laplacian*


$$L_{n}^{\left(s\right)}=D_{n}^{-1/2}\left(D_{n}-W_{n}\right)D_{n}^{-1/2}=I-D_{n}^{-1/2}W_{n}D_{n}^{-1/2},$$

which we obtain by setting p=2,q=3,r=1 . In this framework, the random walk Laplacian is given by p=2,q=2,r=0 .

The graph Laplacian is a finite sample approximation of the Laplace–Beltrami operator on the low-dimensional manifold [57] on which the input data lives; one can often show convergence in the large data limit n→∞ . Thus, when using the graph Laplacian, we do not approximate the manifold immediately, but rather an operator defined on functions on the manifold or, as mentioned above, diffusion defined on the manifold. In the following, we highlight several results that discuss consistency and convergence results that consider the large sample limit of graph Laplacians and graph PDEs.

In the remainder of this section, we will discuss different machine-learning strategies based on the graph Laplacian.

### (b) Spectral clustering

Spectral clustering is an unsupervised learning method for data given on graphs. It uses the eigenvectors of the graph Laplacian to map the graphical data into an Euclidean space where some off-the-shelf clustering technique is applied (typically k -means clustering). There are multiple ways to interpret spectral clustering; we refer to [52] for details.

In [59], the authors study the consistency of spectral clustering in the large data limit. Another method utilizing graph Laplacian eigenvectors is studied in [60]. Using Γ -convergence and optimal transport, the authors of [61] prove consistency and stability results. More works are concerned with the convergence of the eigenvalues and eigenfunctions of the graph Laplacian [53–55].

### (c) Laplace learning

*Laplace learning* and its variants semi-supervised learning strategies. We now introduce the basic idea and several of its variants. The Laplace semi-supervised learning model is concerned with solving the variational problem


(3.1) $$\min_{u:\Omega_{n}\to R}\sum_{i,j=1}^{n}w_{ij}{\left(u\left(x_{i}\right)-u\left(x_{j}\right)\right)}^{2}\text{ subject to }u\left(x_{i}\right)=\ell_{i}(i=1,\ldots ,m),$$

i.e. we aim to find a function u that interpolates the labelled data and otherwise minimizes energy that enforces similarity between function values if the feature inputs are similar. The Euler–Lagrange equation is the following set of equations on the graph G


(3.2){Lnu(xi)=0(i=m+1,…,n),u(xi)=ℓi(i=1,…,m),

where $L_{n}$ is the graph Laplacian defined previously. We refer to this equation as the graph Laplace equation.

Laplace learning was first used in [62]. In [63], it is shown that when the dimension of the data is at least two and the number of labels is fixed, Laplace learning is asymptotically ill-posed in the sense that, as n→∞ , the solution becomes independent of the observed labels $\{\ell_{i}{\}}_{i=1}^{m}$ . In fact, one needs the number of labelled points m to be least $O(n^{d-2/d}(\log n{)}^{2/d})$ for asymptotic well-posedness [64]. Many papers, including [53,65–71], study pointwise convergence of Laplace learning.

### (d) Variations of Laplace learning

When the number of labels is small Laplace learning is degenerate, in the sense that labels are lost [72,73]. The problem is due to lack of regularity of solutions to (3.2). More precisely, solutions to (3.2) can converge to discontinuous limits, and hence the label constraint ( $u(x_{i})=\ell_{i}$ for i=1,…,m ) is not asymptotically well-defined. The consequence is that solutions to (3.2) are converging to constants. A variety of other graph PDEs with better regularity properties have been employed in semi-supervised learning to improve upon Laplace learning. We briefly review a few here.

#### (i) Continuum modelling

**Figure 1.**
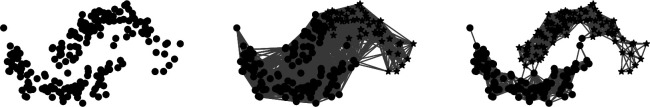
Using large numbers of graph edges can make it harder to refine class boundaries. The raw data (from the two moons dataset) are plotted on the left-hand side. In the middle, we use a graph with many edges, and it becomes difficult to separate the two moons, as illustrated by the poor performance of a phase-separation model. The figure on the right uses a much smaller number of edges, and the same phase-separation model performs much better. A version of this figure appears in [74], which we refer to for details of the phase-separation model.

To illustrate the regularity issues inherent in Laplace learning, we make precise the connection to Sobolev spaces and PDE regularity via taking a large data limit. The assumption we make is that there should be localization in the graph, i.e. nodes are connected that are close in space. This can be motivated from a practical point of view by (i) keeping the weight matrix sparse (which reduces computational cost) and (ii) increasing the resolution, which allows for more refined boundaries between classes (figure 1). We assume (as is common in the literature) that the weights $w_{ij}$ are defined by


$$w_{ij}=\frac{1}{\varepsilon^{d}}\eta \left(\frac{\left\|x_{i}-x_{j}\right\|}{\varepsilon }\right),$$

where η:[0,∞)→[0,∞) is non-increasing, ε>0 is a ‘connectivity radius’ and d is the dimension of the data. If η(t)=1 , when t≤1 , and η(t)=0 otherwise, then $x_{i}$ and $x_{j}$ are connected by an edge if and only if $\|x_{i}-x_{j}\|\leq \varepsilon$ .

The Laplace learning objective functional (with normalization) in (3.1) can be written


$$E_{n}(u)=\frac{1}{n^{2}\varepsilon^{2}}\sum_{i,j=1}^{n}w_{ij}{\left(u\left(x_{i}\right)-u\left(x_{j}\right)\right)}^{2}.$$

It is a straightforward formal calculation to show that if $u\in {\text{C}}^{2}$ and $x_{i}\overset{iid}{∼}\mu \in P(R^{d})$ where μ has density ρ , then (approximately)


$$\begin{array}{rl} E_{n}(u) & =\frac{1}{n^{2}\varepsilon^{2+d}}\sum_{i,j=1}^{n}\eta \left(\frac{\left\|x_{i}-x_{j}\right\|}{\varepsilon }\right){\left(u\left(x_{i}\right)-u\left(x_{j}\right)\right)}^{2}\\ & \approx \frac{1}{\varepsilon^{2+d}}\int_{R^{d}}\int_{R^{d}}\eta \left(\frac{\left\|x-y\right\|}{\varepsilon }\right)(u(x)-u(y){)}^{2}\rho (x)\rho (y)dx\;dy\;\text{ for }n\gg 1\\ & =\int_{R^{d}}\int_{R^{d}}\eta (\|z\|){\left(\frac{u(x)-u(x+\varepsilon z)}{\varepsilon }\right)}^{2}\rho (x)\rho (x+\varepsilon z)dx\;dz\;\text{ using }z=\frac{y-x}{\varepsilon },\\ & \approx \int_{R^{d}}\int_{R^{d}}\eta (\|z\|)(\nabla u(x)\cdot z{)}^{2}\rho^{2}(x)dx\;dz\;\text{ for }\varepsilon \ll 1\\ & =\sigma_{\eta }\int_{R^{d}}\|\nabla u(x){\|}^{2}\rho^{2}(x)dx\;\text{ by isotropy of }z\mapsto \eta (\|z\|), \end{array}$$

where $\sigma_{\eta }=\int_{R^{d}}\eta (\|z\|)z_{1}^{2}\;\text{d}z$ . Hence, as n→∞ and ε→0 , we expect $E_{n}$ to behave like the (weighted) Sobolev ${\text{W}}^{1,2}$ semi-norm. The first variation of Laplace learning we discuss is to use the Sobolev space ${\text{W}}^{1,p}$ , which, for p>d , is a subset of the continuous functions (or more precisely, can be identified with a subset of continuous functions).

#### (ii) Nonlinear graph Laplacians

We start from the p -Dirichlet energy


(3.3) $$\min_{u}\int \|\nabla u(x){\|}^{p}\rho^{2}(x)\;\text{d}x,$$

where p≥1 . Laplace learning is the special case p=2 . Using the energy implied by (3.3) means we work in the Sobolev space ${\text{W}}^{1,p}$ , which, for p>d , is a subset of continuous functions, meaning the variational problem above is well-defined for finite labels, i.e. one can impose the constraint $u(x_{i})=\ell_{i}$ for i=1,…,m .

Treating the simple case where the density is constant (i.e. ρ=1 ), we can follow [75] to infer that a minimizer of (3.3) satisfies the Euler–Lagrange equation


(3.4) $$0=L_{\infty }^{\left(p\right)}(u)=\nabla \cdot \left(|\nabla u{|}^{p-2}\nabla u\right)=|\nabla u{|}^{p-2}\left(\Delta u+(p-2)\Delta_{\infty }u\right).$$

We call $L_{\infty }^{\left(p\right)}$ the *p -Laplacian* and


(3.5) $$L_{\infty }^{\left(g,p\right)}(u):=\Delta u+(p-2)\Delta_{\infty }u$$

the *game-theoretic p -Laplacian*. We define


(3.6) $$\Delta_{\infty }u=\frac{1}{|\nabla u{|}^{2}}\sum_{i,j=1}^{d}(\partial_{i}u)(\partial_{j}u)(\partial_{ij}^{2}u),$$

to be the *infinity Laplacian*. When we set these operators to 0, we obtain PDEs, whose solutions have various advantageous properties. These PDEs allow for the study of complex phenomena in economic and political modelling [76], physics and biology [77], optimal transport [78] and image processing [79], to name a few.

Following [80], the discrete counterparts to (3.3), (3.4), (3.5) and (3.6) are the *discrete p -Dirichlet energy*


(3.7) $$E_{n}^{\left(p\right)}(u)=\frac{1}{n^{2}\varepsilon^{p}}\sum_{i,j=1}^{n}w_{ij}|u(x_{i})-u(x_{j}){|}^{p},$$

the *discrete p -Laplacian* $L_{n}^{\left(p\right)}$


(3.8) $$L_{n}^{\left(p\right)}(u)(x_{i}):=\sum_{j=1}^{n}w_{ij}|u(x_{i})-u(x_{j}){|}^{p-2}(u(x_{i})-u(x_{j})),$$

the *discrete game-theoretic p -Laplacian* $L_{n}^{\left(g,p\right)}$


(3.9) $$L_{n}^{\left(g,p\right)}(u):=\frac{1}{p-1}L_{n}^{\left(2\right)}(u)+\left(1-\frac{1}{p-1}\right)L_{n}^{\left(\infty \right)}(u)$$

and the *discrete graph infinity Laplacian* $L_{n}^{\left(\infty \right)}$


(3.10) $$L_{n}^{\left(\infty \right)}(u)(x_{i})=u(x_{i})-\frac{1}{2}(\min_{x_{j}:w_{ij}>0}u(y)+\max_{x_{j}:w_{ij}>0}u(x_{j})).$$

Note that if the weights in the discrete p -Laplacian (3.8) were replaced by $w_{ij}^{p}$ , then they appear in the following limiting version of the graph infinity Laplacian, then given by


(3.11) $$L_{\infty }u(x_{i}):=\min_{j=1,\ldots ,\ell +n}w_{ij}(u(x_{j})-u(x_{i}))+\max_{j=1,\ldots ,\ell +n}w_{ij}(u(x_{j})-u(x_{i}));$$

see [64] for details. The infinity Laplacian is related to a stochastic zero-sum game called ‘tug of war’. In this game, two players play against each other by trying to move a point towards/away from a target zone; at every turn, determined by a coin toss, one of the players gets to move the point. The game ends when the target zone is reached.

Observe that the p -Laplacian $L_{\infty }^{\left(p\right)}$ and the game-theoretic p -Laplacian $L_{\infty }^{\left(g,p\right)}$ are the same, formally, at the continuum level, but different at the discrete level, as $L_{n}^{\left(p\right)}\neq L_{n}^{\left(g,p\right)}$ [64]. While the p -Laplacian emerged as an optimization problem, the game-theoretic p -Laplacian is related to the ‘tug of war’ game, as it contains the infinity Laplacian in its definition. Numerical approaches to the p -Laplace method have appeared in [81] and a continuum limit analysis (via Γ− convergence) in [73]. The game-theoretic p -Laplacian is analysed in [75] and [80].

We refer to Laplace learning using the infinity Laplacian as *Lipschitz learning*. It is given by


(3.12) $$\begin{array}{r} \left\{ \begin{array}{ll} L_{n}^{\left(\infty \right)}(u)(x_{i})=0 & \left(i=m+1,\ldots ,n\right)\\ u(x_{i})=\ell_{i}\; & (i=1,\ldots ,m). \end{array}\right. \end{array}$$

Lipschitz learning has first been introduced in [82], and continuum limits have been investigated in [83–85]. The methodology is particularly useful when class imbalance is an issue, i.e. when certain labels appear much more frequently than others [86]. Another important special case is the *discrete 1 -Dirichlet energy*


$$E_{n}^{\left(1\right)}(u)=\sum_{i,j=1}^{n}w_{ij}|u(x_{i})-u(x_{j})|,$$

which is equivalent to the graph total variation seminorm or the graph cut energy. This graph total variation functional has attracted substantial interest (e.g. [87–90]) and forms the basis for several alternative graph-based methods such as Ginzburg–Landau functionals (see the section on other approaches below).

#### (iii) Fractional Laplacians

Another way to increase the regularity (in particular to achieve continuity) is to ‘increase the number of derivatives’. Formally, this means that instead of working in ${\text{W}}^{1,p}$ , one works in ${\text{W}}^{k,2}$ . If $k>\frac{d}{2}$ , then ${\text{W}}^{k,2}$ is a subset of the continuous functions. Derivatives are defined at the discrete level through an eigenbasis expansion. In particular, if $\left(\lambda_{i}^{\left(n\right)},q_{i}^{\left(n\right)}\right)$ *,* for i=1,…,n *,* are eigenpairs of the Laplacian $L_{n}^{\left(2\right)}$ then one can define the objective functional


$$F_{n}^{\left(k\right)}(u)=\sum_{i=1}^{n}{\left[\lambda_{i}^{\left(n\right)}\right]}^{k}{\left\langle q_{i}^{\left(n\right)},u\right\rangle }_{n},$$

where $\left\langle u,v\rangle_{n}=1/n\sum_{x\in \Omega_{n}}u(x)v(x\right)$ is the discrete inner product. When k=1 , then one recovers Laplace learning as $F_{n}^{\left(1\right)}(u)=cE_{n}^{\left(2\right)}(u)$ for some constant c independent of u . The method was proposed in [91], with subsequent analysis in [92–95].

### (e) Other approaches

A number of other approaches have been proposed in the literature. *Reweighted Laplace Learning* [96,97] increases weights around labels in order to increase the regularity only at labels using, for example, the objective


(3.13) $$\sum_{i=m+1}^{n}\sum_{j=1}^{n}w_{ij}(u(x_{i})-u(x_{j}){)}^{2}+\frac{n-m}{m}\sum_{i=1}^{m}\sum_{j=1}^{n}w_{ij}(\ell_{i}-u(x_{j}){)}^{2}.$$

*Poisson Learning* [98] considers the behaviour of Laplace learning at very low label rates and shows that this approximates a Poisson equation. The derived Poisson equation does not have pointwise constraints and therefore does not suffer the same degeneracy as Laplace learning. The method is often state-of-the-art when the label rate is extremely low. Other PDEs and related techniques that have found use in graph-based learning include *Eikonal equations* [99–101], *diffusion maps* [65], *Ginzburg–Landau functionals* [102–105], *Mumford–Shah functionals* [106], *graph cuts* (such as Cheeger and ratio cuts) [107–109] and *Dirichlet partitions* [110]. We refer to the book by van Gennip & Budd [111] for a more detailed introduction to semi-supervised learning with nonlinear PDEs.

The PDE-based techniques presented throughout this section are closely related to the variational techniques for inverse problems discussed in Section 2 with regularizers given through differential operators on graphs. Similarly, Bayesian techniques can be translated to the graphical setting. Gaussian measures with Whittle–Matérn covariance operators on graphs have been studied by [112–114]. Moreover, Bertozzi *et al*. [115] investigated the use of Ginzburg–Landau energies as priors in Bayesian classification on graphs.

## 4. Partial differential equations and neural networks

PDEs have appeared in the following two ways in the neural network community: (i) PDEs for neural networks and (ii) neural networks for PDEs. The latter refers to creating neural networks to solve PDEs, while the former refers to using ideas from PDE theory to design and analyse neural networks. We focus on the former; for the latter, we refer to the 2022 survey [116] and to Volume 381, Issue 2260 and Volume 382, Issue 2264 of the *Philosophical Transactions of the Royal Society A*. We discuss three aspects of PDEs for neural networks: the construction of residual neural networks (ResNets) from neural ordinary differential equations (ODEs), graph neural diffusions from graph heat equations and flows at the foundation of transformers and large language models.

### (a) From perceptron to residual neural network

Neural networks have been around since at least the 1950s, and they have undergone several waves of popularity in that time. Due to success in classification and imaging tasks, they are strongly back in fashion. The size of neural networks has increased as computational resources have developed, and the size of neural networks is now typically larger than it was 50+ years ago. Larger networks can be seen as more desirable because they (in theory) allow a better approximation. However, there are often problems in the training of large neural networks.

The vanilla neural network is simply an iteration of a linear functional and a nonlinear activation function. In particular, we let $X_{k}\in R^{d}$ be the vector, which describes the state of d neurons in layer k (for simplicity, let us assume all layers have the same number of neurons apart from the very last layer). We are given an input $X_{0}=x$ , and we wish to estimate a label $\ell \approx u(x)\in R^{m}$ . In a neural network with K layers, we define the perceptron by


$$X_{k}=\sigma_{k-1}(A_{k-1}X_{k-1}+b_{k-1})\;\;k=1,\ldots ,K-1$$

and $u(x)=\sigma_{K}(A_{K-1}X_{K-1}+b_{K-1})$ where $A_{k}\in R^{d\times d},\;b_{k}\in R^{d}$ for k=0,…,K−2 and $A_{K-1}\in R^{d\times m},\;b_{K-1}\in R^{m}$ are learnable parameters and $\sigma_{k}$ ( k=0,…,K−1 ) is the activation function acting pointwise. A neural network is deep when K is large. In theory, K being large should allow a better approximation of $\ell_{i}\approx u(x_{i})$ (since we have more degrees of freedom); however, this is often not the case with the performance of the neural network decreasing with the number of layers. This is often called the degradation problem [117,118]. The increase in error happens during training and, therefore, cannot be attributed to overfitting. A second problem noticed for the deep vanilla neural network is the problem of exploding/vanishing gradients during training (e.g. [119–122]). We attempt to explain this with an example below; essentially what happens is that the parameters we are learning, $A_{k}$ and $b_{k}$ , either hardly change (learning is very slow) or change too quickly (learning is unstable).

Ideas from PDEs/ODEs have attracted a lot of attention. This allows one to introduce a model into the network, which makes it possible to consider limits (e.g. [123–125]), design better architectures by transferring ideas from well-understood PDEs/ODEs (e.g. [126]) and apply well-established analytical tools to understand neural networks (e.g. [127]). The first (as far as the authors are aware) to make the connections between PDEs/ODEs and neural networks precise were Haber & Ruthotto [128] and E [129]. The idea is to add a so-called skip-connection between labels and so model the *residual*. In particular, the architecture can then be written


$$X_{k}=X_{k-1}+\sigma_{k-1}(A_{k-1}X_{k-1}+b_{k-1})\;\;k=1,\ldots ,K-1.$$

If we add a scaling term in front of the activation (or absorb it into the constants $A_{k}$ and $b_{k}$ if the activation is 1-homogenous), then we see that this is just a time discretization of the ODE


$$\dot{X}(t)=\sigma_{t}(A(t)X(t)+b(t))\;\;t\in [0,1].$$

This defines the *ResNet* class.

To compare (synthetically) ResNet with the vanilla neural network, we consider a cost function $C(\ell ,\ell^{†})$ that measures the difference between the predicted label ℓ and the true label $\ell^{†}$ . If we ignore the activation function σ and the bias terms $b_{k}$ , then we have $X_{K}=A_{K-1}A_{K-2}\cdots A_{k}X_{k}$ . So, since our predicted label is $X_{K}$ , we have


$$\frac{\partial }{\partial A_{k}}C(X_{K},\ell^{†})=\nabla_{1}C(X_{K},\ell^{†})A_{K-1}\ldots A_{k+1}X_{k},$$

where $\nabla_{1}$ is the derivative with respect to the first argument of C . Now, if each $A_{k}$ is roughly equal in magnitude, say $A_{k}\approx A$ , we have that the gradient of the cost is either exponentially increasing or decreasing with respect to the number of layers. In particular,


$$\frac{\partial }{\partial A_{k}}C(X_{K},\ell^{†})\approx \nabla_{2}C(X_{K},\ell^{†})A^{K-k}X_{k}.$$

The only way this has a non-divergent limit is if det⁡(A)=±1 . Formally, this is restricted to rotations and reflections.

On the other hand, in ResNet, with the same formal calculation, we obtain


$$X_{K}=\left(\mathrm{Id}+A_{K}\right)\ldots \left(\mathrm{Id}+A_{k}\right)X_{k}$$

and, therefore,


$$\frac{\partial }{\partial A_{k}}C\left(X_{K},\ell^{†}\right)=\nabla_{1}C\left(X_{k},\ell^{†}\right)\left(\mathrm{Id}\;+A_{K}\right)\cdots \left(\mathrm{Id}\;+A_{k+1}\right)X_{k}.$$

Now, if each $A_{k}$ is roughly equal (as before $A_{k}\approx A$ ), then we expect $\frac{\partial }{\partial A_{k}}C(X_{K},\ell^{†})\approx \exp((K-k)A)$ . Renormalizing A↦A/K , we get that the derivative is bounded. Note that after the normalization, we do not need to make any assumptions on A to get a derivative that is neither exponentially increasing nor decreasing.

Of course, the toy example is very simple and ignores effects from the activation function (for example). Nevertheless, the formal reasoning agrees with empirical observations in that ResNets scale better to deep layers than the vanilla neural network.

ResNet uses an explicit Euler discretization of a first-order ODE. This opened the door to trying alternative discretizations and different ODE models to motivate new architectures. For example, one can train with a method that involves solving the adjoint ODE (via classical numerical analysis techniques) avoiding backpropagation [130]. Beyond explicit Euler, one can use symplectic discretization (e.g. [128]) or the linear multi-step method [131], which uses the discretization


$$X_{k}=(1-\tau_{k})X_{k-1}+\tau_{k}X_{k-2}+\sigma_{k-1}(A_{k-1}X_{k-1}+b_{k-1}).$$

Furthermore, connections with optimal control problems give access to well-understood theory, for example, Hamilton–Jacobi–Bellman equations and Pontryagin’s maximum principle [132]. In turn, this opens up new training paradigms with error estimates [133].

ResNet has inspired new architectures using the stability of the underlying ODE (e.g. [128,134]). To avoid vanishing/exploding gradients in training, Haber & Ruthotto [128] argue that the eigenvalues of $A_{k}$ should be imaginary. They proposed enforcing antisymmetric in the $A_{k}$ and a new second-order ODE model based on a Hamiltonian system. Architectures designed to control computational cost (in particular memory) have been proposed in [134]. Several architectures, such as PolyNet, FractalNet and ResNet, were unified in [131] as different discretizations of different ODEs. Second-order methods (effectively using the ODE $\ddot{X}(t)+\gamma \dot{X}(t)=\sigma_{t}(X(t))$ ) have been proposed in [135]. These are called heavy-ball neural ordinary differential equations and have the advantage over first-order systems in that the adjoint is also a heavy-ball neural ordinary differential equation, which reduces the number of function evaluations (hence making these methods more computationally efficient).

ODE models have also appeared in generative models [136]. PDE models have been used to define convolutional neural networks [126,137]. Stochastic models (i.e. stochastic differential equations) have also been used to inject noise (e.g. [131]), as well as analyse mean-field limits (when the number of training samples converges to infinity) of the idealized time-continuous deep limit [138].

### (b) Graph neural diffusions

Graph neural networks have been developed for situations where the data are not Euclidean, i.e. they comes with some manifold structure. We again represent this structure via a graph with nodes $\Omega_{n}=\{x_{i}{\}}_{i=1}^{n}$ and weight matrix $W=(w_{ij}{)}_{i,j=1,\ldots ,n}$ . Similarly to the graph Laplacian, we can define gradient and divergence operators by


$$\begin{array}{c} \nabla_{n}:L^{0}\left(\Omega_{n}\right)\to L^{0}\left(\Omega_{n}\times \Omega_{n}\right)\\ \left[\nabla_{n}u_{n}\right]\left(x_{i},x_{j}\right)=u_{n}\left(x_{i}\right)-u_{n}\left(x_{j}\right) \end{array}$$

and


$$\begin{array}{rl} {\mathrm{div}}_{n}:L^{0}\left(\Omega_{n}\times \Omega_{n}\right) & \to L^{0}\left(\Omega_{n}\right)\\ \left[{\mathrm{div}}_{n}U_{n}\right]\left(x_{i}\right) & =\sum_{j=1}^{n}w_{ij}U_{n}\left(x_{j}\right). \end{array}$$

In the above, we write ${\text{L}}^{0}$ as the space of functions (on the given domain). We define the inner products by


$$\begin{array}{rl} \langle u_{n},v_{n}\rangle_{n} & =\sum_{i=1}^{n}u_{n}(x_{i})v_{n}(x_{i})\\ {\left\langle \left\langle U_{n},V_{n}\right\rangle \right\rangle }_{n} & =\frac{1}{2}\sum_{i,j=1}^{n}w_{ij}U_{n}(x_{i},x_{j})V_{n}(x_{i},x_{j}). \end{array}$$

Then, the gradient and divergence operators are adjoint when restricted to the set of antisymmetric edge functions, i.e.


$${\left\langle u_{n},{\mathrm{div}}_{n}V_{n}\right\rangle }_{n}={\left\langle \left\langle \nabla_{n}u_{n},V_{n}\right\rangle \right\rangle }_{n}$$

for all $u_{n}:\Omega_{n}\to R$ and $V_{n}:\Omega_{n}\times \Omega_{n}\to R$ with $V_{n}(x_{i},x_{j})=-V_{n}(x_{j},x_{i})$ . We can define the graph Laplacian by $L_{n}={\text{div}}_{n}\circ \nabla_{n}$ (note that by convention, the graph Laplacian is positive semi-definite unlike the usual Laplacian, $\Delta u=\sum_{i=1}^{d}\partial^{2}u/\partial x_{i}^{2}$ , which is negative semi-definite). The analogue of the heat equation in Euclidean settings ( ∂u/∂t(t,x)=Δu(t,x) ) on graphs is $\partial u/\partial t(t,x)=L_{n}u(t,x)$ (ignoring the change of sign).

To unify many graph neural network architectures, Chamberlain *et al*. [139] proposed a framework, GRAND, based on diffusion equations. Their idea was to use


(4.1) $$\frac{\partial u}{\partial t}(t,x)={\mathrm{div}}_{n}\left[G(u(t,\cdot ),t)\nabla_{n}u(t,\cdot )\right](x),\;t\geq 0,x\in \Omega_{n}$$

for some functional G . The choice of parametrization for G and the type of time discretization determines the graph neural network architecture. To help the model scale to deep layers—the diffusivity is likely responsible for the oversmoothing phenomena where u(t,⋅) becomes increasingly constant for large t —a source term was added in [140].

The previous architecture, based on (4.1), is a parameterization of a flow. In fact, the heat equation (if G is the identity) is the ${\text{L}}^{2}$ gradient flow of the Dirichlet energy


$$E_{n}(u)=\frac{1}{4}\sum_{i,j=1}^{n}w_{ij}{\left|u\left(x_{i}\right)-u\left(x_{j}\right)\right|}^{2}.$$

Therefore, GRAND can be seen as a parametrization of a gradient flow (although we are not claiming it is a gradient flow). The idea in [141] was to parameterize the energy rather than the gradient flow. If we take a more general energy of the form


(4.2) $$F_{n}(u)=\sum_{i=1}^{n}u{\left(x_{i}\right)}^{⊤}Au\left(x_{i}\right)-\langle u,Bu\rangle_{n}-\langle u,C\ell \rangle_{n},$$

where we assume $u:\Omega_{n}\to R^{k}$ , $A\in R^{k\times k}$ , $B\in R^{d\times d}$ and $C\in R^{d\times d}$ are learnable parameters. The advantage of parameterizing the energy is that one now has interpretability. The first term in (4.2) can be understood as a damping term, the second term is an attraction/repulsion term and the last term incorporates known labels. In particular, the second term is either driving values $u(x_{i})$ and $u(x_{j})$ towards each other or apart. Once the parameters A , B and C are learnt, one is able to interpret the model. For further details on Graph Neural Diffusions, in particular their drawbacks, their relationship with continuous dynamics, and numerical schemes, see [142].

### (c) Transformers

Large language models (LLMs) are currently very popular, driven by the success of programs such as ChatGPT (OpenAI), Llama 3 (Meta), Gemini and Gemma (Google/DeepMind) and Phi-3 (Microsoft). Large language models are designed for natural language processing, for example, language generation. In language generation, the model should be able to respond to some input, giving the appearance of being able to hold a conversation or answer a question.

Before applying an LLM to a prompt, some preprocessing converts the prompt to a set of tokens, which is a way to vectorize, compress and standardize the input, i.e. convert the input to a numerical data type. The building blocks for LLMs are transformers that can be seen from a dynamical systems viewpoint as a way to model the flow of tokens from the input to a desired output. More precisely, [143,144] showed that the evolution of tokens through a transformer is given by a convection–diffusion equation. If we consider n d -dimensional tokens as inputs, ${\bar{x}}_{i}\in R^{d}$ ( i=1,…,n ), the dynamics can be described in a time-continuous setting by


(4.3) $$\frac{dx_{i}}{\;dt}(t)=P_{x_{i}(t)}(w(t)\sigma \left(\sum_{h=1}^{H}\sum_{j=1}^{n}A_{ijh}(t)\left(V_{h}(t)x_{j}(t)+b_{h}(t)\right)\right),\;x_{i}(0)={\bar{x}}_{i}(0),$$

where $P_{x}y$ is the projection of $y\in S^{d-1}$ onto the tangent space at $x\in S^{d-1}$ (where $S^{d-1}$ is the unit sphere in $R^{d}$ ), H is the number of attention heads (see [145]), $A_{ijh}(t)$ measures the interaction between tokens i and j in the h th head, $V_{h}$ is called the value matrix, w is a weight term and $b_{h}$ is a bias term. Here, we have simplified the interaction, which usually takes the form


$$A_{ijh}(t)=\frac{e^{\beta \langle Q_{h}(t)x_{i}(t),K_{h}(t)x_{j}(t)\rangle }}{Z_{\beta ih}(t)},\;Z_{\beta ih}(t)=\sum_{j=1}^{n}e^{\beta \langle Q_{h}(t)x_{i}(t),K_{h}(t)x_{j}(t)\rangle },$$

where $Q_{h}$ and $K_{h}$ are called the query and key matrices, respectively. A simple model for transformers is a discretization of (4.3).

One can take this a step further and write (4.3) in terms of a continuity equation:


(4.4) $$\frac{\partial \mu_{n}}{\partial t}+\mathrm{div}\left(X\left[\mu_{n}\right]\mu_{n}\right)=0\;\text{ on }[0,+\infty )\times S^{d-1},\;\mu_{n}(0)=\frac{1}{n}\sum_{i=1}^{n}\delta_{{\bar{x}}_{i}},$$

where $\mu_{n}(t)=\frac{1}{n}\sum_{i=1}^{n}\delta_{x_{i}(t)}$ and


$$X[\mu ](t,x)=P_{x}\left(w(t)\sigma \left(\sum_{h=1}^{H}\int A_{xyh}(t)\left(V_{h}(t)y+b(t)\right)d\mu (y)\right)\right).$$

If one simplifies the model further so that we can assume


$$X[\mu ](t,x)=P_{x}\left(\int e^{\beta \langle y,x\rangle }y\;d\mu (y)\right),$$

then $\mu_{n}(t)$ following (4.4) is a Wasserstein gradient flow for the energy [146]


$$E_{\beta }(\mu )=\frac{1}{2\beta }\iint e^{\beta \langle x,y\rangle }d\mu (x)d\mu (y).$$

The dynamical formulation of transformers has attracted many follow-up works, for example [147–151].

## 5. An editorial foreword

The theme issue ‘Partial Differential Equations in Data Science’ presents 12 research articles spanning most aspects of PDEs appearing in data science. At this point, research in this area usually appears in scientific journals covering applied and numerical analysis, probability or statistics or at machine learning conferences. Our goal was especially to open a venue in which these results are collected in context and presented jointly: it enhances visibility within the community, supports collaboration and acts as an entry point for new members of the community.

The theme issue covers many aspects of PDEs in data science: gradient flows in multiplex networks, spectral total variation learners, fractional Brownian motions on the sphere, emulation of dynamical systems through geometric convolutions, attraction–repulsion swarming for dimension reduction, neural PDE solvers, diffusion models and their Fokker–Planck equations, infinite-dimensional sampling in the analysis of PDEs, Wasserstein gradient flows, evolutionary dynamics in Bayesian inference, consensus-based bi-level optimization in federated learning and mean-field models arising in transformers.

A theme issue in the *Philosophical Transactions* is an opportunity for a research community to showcase current research to a wide range of readers. The organization of this theme issue was a great pleasure for us.

## Data Availability

This article has no additional data.
